# Bortezomib Treatment Modulates Autophagy in Multiple Myeloma

**DOI:** 10.3390/jcm9020552

**Published:** 2020-02-18

**Authors:** Giuseppe Di Lernia, Patrizia Leone, Antonio Giovanni Solimando, Alessio Buonavoglia, Ilaria Saltarella, Roberto Ria, Paolo Ditonno, Nicola Silvestris, Lucilla Crudele, Angelo Vacca, Vito Racanelli

**Affiliations:** 1 Department of Biomedical Sciences and Human Oncology, Unit of Internal Medicine “Guido Baccelli”, University of Bari Medical School, 70124 Bari, Italy; giuseppe.dilernia@uniba.it (G.D.L.); patrizia.leone@uniba.it (P.L.); antoniogiovannisolimando@gmail.com (A.G.S.); alessio.buonavoglia85@gmail.com (A.B.); ilaria.saltarella@libero.it (I.S.); roberto.ria@uniba.it (R.R.); n.silvestris@oncologico.bari.it (N.S.); lucillacrudele@gmail.com (L.C.); angelo.vacca@uniba.it (A.V.); 2 IRCCS Istituto Tumori “Giovanni Paolo II”, 70124 Bari, Italy; paolo.ditonno@fastwebnet.it

**Keywords:** autophagy, angiogenesis, multiple myeloma, plasma cells, endothelial cells, drug resistance, bortezomib

## Abstract

Although the introduction of bortezomib as a therapeutic strategy has improved the overall survival of multiple myeloma (MM) patients, 15–20% of high-risk patients do not respond to bortezomib over time or become resistant to treatment. Therefore, the development of new therapeutic strategies, such as combination therapies, is urgently needed. Methods: Given that bortezomib resistance may be mediated by activation of the autophagy pathway as an alternative mechanism of protein degradation, and that an enormous amounts of misfolded protein is generated in myeloma plasma cells (PCs), we investigated the effect of the simultaneous inhibition of proteasome by bortezomib and autophagy by hydroxychloroquine (HCQ) treatment on PCs and endothelial cells (ECs) isolated from patients with monoclonal gammopathy of undetermined significance (MGUS) and MM. Results: We found that bortezomib combined with HCQ induces synergistic cytotoxicity in myeloma PCs whereas this effect is lost on ECs. Levels of microtubule-associated protein light chain beta (LC3B) and p62 are differentially modulated in PCs and ECs, with effects on cell viability and proliferation. Conclusions: Our results suggest that treatment with bortezomib and HCQ should be associated with an anti-angiogenic drug to prevent the pro-angiogenic effect of bortezomib, the proliferation of a small residual tumor PC clone, and thus the relapse.

## 1. Introduction

Multiple myeloma (MM, see Table 1 for a list of abbreviations and acronyms used) is a malignant proliferation of monoclonal plasma cells (PCs) with a strong tropism for bone and bone marrow. It is preceded by a preneoplastic phase, termed monoclonal gammopathy of undetermined significance (MGUS) [1]. Among PC dyscrasias, MM is the second most frequent hematological malignancy, with 32,110 cases predicted to have occurred in the USA in 2019 [2]. Although the majority of patients respond to initial therapy, most will ultimately suffer disease relapse due to the proliferation of resistant tumor cells [3,4]. However, with the recent introduction of immunomodulatory drugs and proteasome inhibitors, the prognosis of MM patients has substantially improved [5,6]. Bortezomib (Velcade, formerly PS-341), a reversible inhibitor of the chymotrypsin-like activity of the proteasome [7], directly inhibits the proliferation of myeloma cells, induces their apoptosis, reverses drug resistance, and affects myeloma cell interactions with the bone marrow microenvironment by blocking cytokine circuits, cell adhesion, and angiogenesis in vivo [8,9,10,11]. The effectiveness of bortezomib in the treatment of patients with relapsed refractory MM was first demonstrated in a multicenter, open-label, nonrandomized phase 2 study [12] and then confirmed in a large randomized phase 3 trial that compared bortezomib with high-dose dexamethasone in MM patients who relapsed after one to three other therapies [13]. Although bortezomib is a valid alternative for MM patients, relapse due to bortezomib-resistance is inevitable and the disease, at present, remains incurable. In MM patients, each different plasma cell sub-clone mimics a tumor-initiating niche. Thus, while bortezomib eliminates bortezomib-sensitive tumor plasma cell sub-clones, it also promotes the expansion of pre-existing bortezomib-resistant sub-clones, leading to relapse [14,15,16]. Consequently, the development of new therapeutic strategies, such as combination therapies, that overcome bortezomib resistance is urgently needed [17,18].

Bortezomib resistance may be mediated by the activation of the autophagy pathway, as an alternative mechanism of protein degradation [19,20]. Autophagy is a catabolic process that is well conserved among all mammals because it modulates homeostasis, by clearing and recycling damaged or useless cell constituents [21]. Due to the enormous amounts of misfolded protein generated in malignant PCs, autophagy modulation becomes a pro-survival mechanism in the event of proteasomal inhibition. Previous studies demonstrated that simultaneous proteasome and autophagy inhibition results in the accumulation of ubiquitinated proteins and thus in cytotoxicity [20,22,23]. Moreover, autophagy inhibition enhances the efficacy of many anticancer drugs, both in cell lines and in animal models [24,25,26,27,28], and synergistically increases bortezomib cytotoxicity in in vitro models of myeloma [29,30], colon carcinoma [31], and hepatocarcinoma [25]. Hydroxychloroquine (HCQ) is a well-known inhibitor of autophagy [32,33,34] and is commonly used to treat autoimmune diseases [35,36,37]. A therapeutic strategy based on the combined targeting of proteasomal and autophagic protein degradation was investigated in a phase I trial in which 25 patients with relapsed/refractory myeloma were treated with bortezomib and HCQ [38]. The results demonstrated that this drug combination is therapeutically feasible, well-tolerated and potentially effective in improving outcome in MM patients. Out of 22 patients evaluable for response, 3 (14%) had very good partial responses, 3 (14%) had minor responses, and 10 (45%) had a period of stable disease, suggesting an enhanced efficacy of the combined drugs vs. bortezomib alone [38].

**Figure 1:**
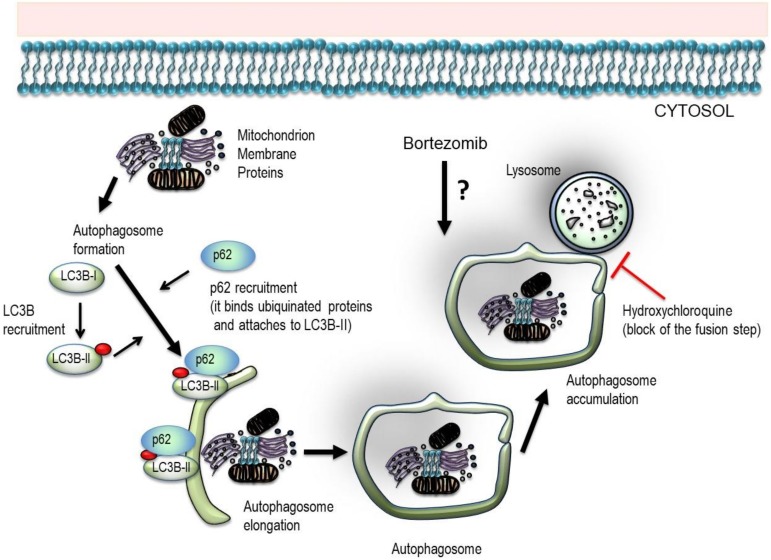
Schematic overview of the experimental design. Cytoplasmic cargo material including mitochondria, membrane, and unfolded proteins is engulfed by a double-membrane vesicle to form and elongate into an autophagosome. Autophagy related (ATG) proteins create two “ubiquitin-like conjugation systems” that catalyze the conversion of LC3B-I to its phosphatidylethanolamine (PE)-conjugated LC3B-II form. LC3B-II protein is recruited to the autophagosome and P62 along with the attached ubiquitinated proteins bind to LC3B-II protein. The complex LC3B-II-p62 is then incorporated into the autophagosome membrane, where it serves as a docking site of adaptor proteins and bound cargo. The mature autophagosome then fuses with the lysosome and forms an autolysosome, leading to cargo degradation and recycling of nutrients and metabolites. Hydroxychloroquine inhibits autophagosome–lysosome fusion and degradation. Thus, HCQ treatment promotes intracellular autophagosome accumulation that correlates with LC3B-II and p62 levels.

In the present study, we investigated the effect of bortezomib and HCQ treatment on PCs and endothelial cells (ECs) isolated from patients with MGUS and MM (Figure 1). We show that bortezomib combined with HCQ induces synergistic cytotoxicity in myeloma PCs, whereas this effect is lost on bone marrow ECs.

## 2. Results

To determine the effects of bortezomib modulation on autophagic flux in PCs and ECs from MGUS and MM patients, we first determined the optimal HCQ concentration and treatment time to achieve total autophagy inhibition, characterized by autophagosome accumulation and pathway saturation, in both cell types. RPMI 8226 plasma cell lines, as well as ECs from MGUS (MGECs) and MM (MMECs) patients, were treated with increasing concentrations of HCQ (0.5–500 µM) for 0, 2, 4, 6, and 24 h (Figure S1).

The autophagic flux was monitored by measuring the levels of microtubule-associated protein light chain beta (LC3B)-II and p62 on a western blot. LC3B-II is associated with both the outer and inner membranes of the autophagosome and is thus used as a marker for these organelles [39]. p62 (also known as SQSTM1/sequestome 1) is a cytoplasmic protein that shuttles ubiquitinated proteins to autophagosomes. It is integrated into autophagosomes through direct binding to LC3B-II and is efficiently degraded by autophagy [40]; thus, total cellular expression levels of p62 inversely correlate with autophagic activity [39].

The results of the LC3B-II and p62 measurements indicated that 100 μM of HCQ was the optimal concentration and 24 h the optimal treatment time to achieve autophagy saturation. Under these conditions, LC3B-II protein levels were 6-fold higher than in the control RPMI 8226 cells (Figure S1A,B), while in the former p62 levels were reduced by half (Figure S1A,C).

In ECs treated with 100 μM HCQ for 24 h, LC3-II (membrane-bound form of LC3) levels increased 3.5-fold in both MGECs and MMECs whereas p62 levels were reduced by 60% and 80%, respectively, indicating saturation of the autophagic flux (Figure S1D–F).

**Figure 2:**
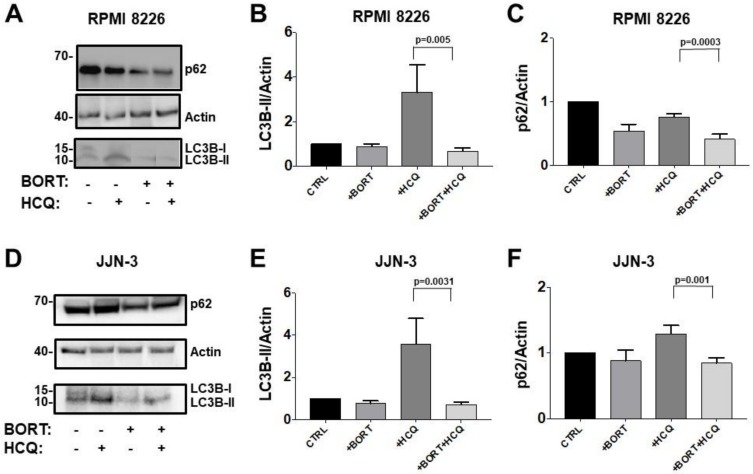
Bortezomib and hydroxychloroquine combination decreases autophagosome formation acting as a downregulator of autophagic flux in plasma cells (PCs). RPMI 8226 (**A**) and JJN-3 (**D**) cells were treated with or without bortezomib (10 nM), hydroxychloroquine (HCQ, 100 uM), or with both drugs for 24 h, followed by immunoblotting analysis to determine LC3B-II and p62 expression levels under each condition. Densitometric analysis of RPMI 8226 (**B**,**C**) and JJN-3 (**E**,**F**) lysates for LC3B-II (**B**,**E**) and p62 (**C**,**F**) expression. The results are expressed as fold-change normalized to the β-actin level and relative to the control. Mann–Whitney U test.

**Figure 3:**
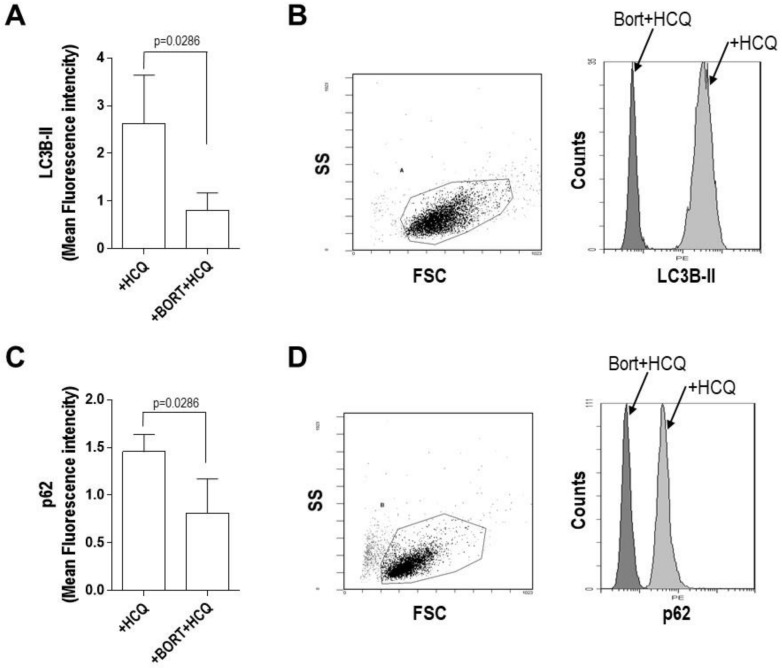
Bortezomib and hydroxychloroquine combination downregulates the autophagic flux in plasma cells (PCs). Changes in LC3B-II and p62 levels upon treatment with hydroxychloroquine (HCQ, 100 uM) alone or both bortezomib (10 nM) and HCQ for 24 h, determined by flow cytometry. Mean Fluorescence intensity (**A**,**C**) and representative plots (**B**,**D**) of primary plasma cells isolated from MM patients (*n* = 6). Mann–Whitney U test.

To investigate the mechanism by which bortezomib modulates the autophagic flux in PCs and ECs, bortezomib treatment was introduced after autophagic flux saturation in response to HCQ had been reached in both cell types. Thus, MM cells were treated with 10 nM bortezomib for 24 h [41]. An increase in LC3B-II levels under saturation in response to HCQ treatment was interpreted as positive modulation of autophagic flux and a decrease in LC3B-II levels as negative modulation. Treatment of the RPMI 8226, JJN-3 plasma cell lines, and bone marrow PCs isolated from MM patients with bortezomib and HCQ resulted in a strong reduction of LC3B-II levels compared with cells treated with HCQ alone, from 3.46 ± 1.45 to 0.77 ± 0.16 in RPMI 8226 cells (*p* = 0.005) (Figure 2A,B), from 3.58 ± 0.6 to 0.69 ± 0.07 in JJN-3 cells (*p* = 0.0031) (Figure 2D,E), and from 2.63 ± 1.00 to 0.81 ± 0.36 in primary MM PCs (*p* = 0.0286) (Figure 3A,B). These observations suggested that bortezomib decreases autophagosome formation acting as a downregulator of the autophagic flux in PCs, a finding confirmed by the analysis of p62 levels. In fact, in cells treated with a combination of bortezomib and HCQ there was a strong reduction in p62 levels compared with cells treated with HCQ alone, from 0.76 ± 0.05 to 0.41 ± 0.07 (*p* = 0.0003) in RPMI 8226 cells, from 1.29 ± 0.12 to 0.84 ± 0.07 (*p* = 0.001) in JJN-3 cells (Figure 2C,F), and from 1.45 ± 0.18 to 0.81 ± 0.36 in primary MM PCs (*p* = 0.0286) (Figure 3C,D).

Taken together, these findings suggested that bortezomib treatment enhances the inhibition of the autophagy pathway that occurs in myeloma PCs in response to HCQ. Bortezomib downregulates the autophagic flux decreasing autophagosome formation. Thus, the additional blockade of autophagosome–lysosome fusion by HCQ determines a reduction in autophagosome accumulation. This results in a decrease of p62 levels that become lower than those observed in cells treated with HCQ alone.

**Figure 4:**
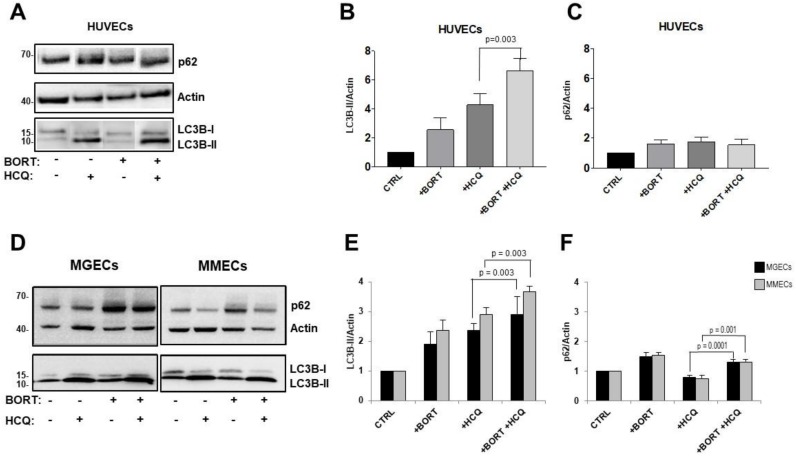
Bortezomib and hydroxychloroquine combination upregulates autophagosome formation in endothelial cells (ECs). (**A**) Human umbilical vein endothelial cells (HUVECs), (**D**) ECs from monoclonal gammopathy of undetermined significance (MGECs, *n* = 9) and ECs from multiple myeloma (MMECs, *n* = 11) were treated with or without bortezomib (10 nM), HCQ (100 uM), or with both drugs for 24 h, followed by immunoblotting to determine LC3B-II and p62 levels under each condition. (**B**–**F**) Densitometric analysis of HUVEC (**B**,**C**), MGEC (black bars), and MMEC (gray bars) (**E**,**F**) lysates for LC3B-II (**B**,**E**) and p62 (**C**,**F**) expression. The results are expressed as fold-change normalized to the β-actin level and relative to the control. Mann–Whitney U test.

By contrast, the opposite results were obtained in human umbilical vein endothelial cells (HUVECs) and bone marrow ECs isolated from MGUS and MM patients (Figure 4). When cells were treated with both bortezomib and HCQ, LC3B-II levels increased compared with cells treated with HCQ alone, up to 6.63 ± 0.49 (*p* = 0.003) in HUVECs (Figure 4A,B), 2.948 ± 0.57 in MGECs (*p* = 0.003), and 3.66 ± 0.62 (*p* = 0.003) in MMECs (Figure 4D,E). The effect was greater in ECs from MM patients than in those from MGUS patients, although the difference was hardly significant (Figure 4E).

Treatment with both drugs led to a similar increase in p62 levels, from 0.78 ± 0.08 to 1.30 ± 0.04 (*p* = 0.001) in MGECs and from 0.75 ± 0.05 to 1.31 ± 0.04 (*p* = 0.0001) in MMECs compared with cells treated with HCQ alone (Figure 4D,F). Treatment of HUVECs did not cause an increase in p62 levels (Figure 4A,C).

Overall, these results indicated that bortezomib stimulates autophagic flux and consequently the autophagosome formation in ECs. This effect, when combined with that of HCQ (i.e., the blockade of autophagosome–lysosome fusion), causes the accumulation of many autophagosomes along with the accumulation of p62 protein in the cytoplasm of ECs.

**Figure 5:**
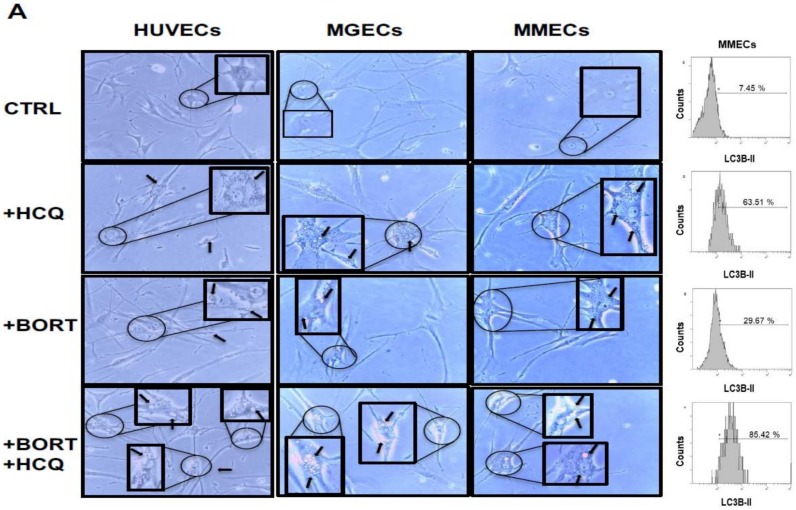
Bortezomib and hydroxychloroquine combination increases autophagosome number in ECs. (**A**) HUVECs, MGECs (*n* = 4), and MMECs (*n* = 4) were treated with HCQ (100 uM), bortezomib (10 nM), or both drugs. Representative photomicrographs (on the left) and flow cytometry plots (on the right) of four independent experiments are shown. Black arrows indicate autophagosome vacuoles (magnification 20×).

Autophagosome accumulation in the cytoplasm of ECs was then examined by conventional light microscopy (Figure 5). HUVEC, MGECs, and MMECs were treated with 10 nM bortezomib and 100 µM HCQ, either alone or in combination, for 24 h. The number and size of the autophagosomes (dark arrows) were progressively enhanced in control, bortezomib alone, HCQ alone, and combined treatment (Figure 5), indicative of the positive modulation of the autophagic pathway by bortezomib.

**Figure 6:**
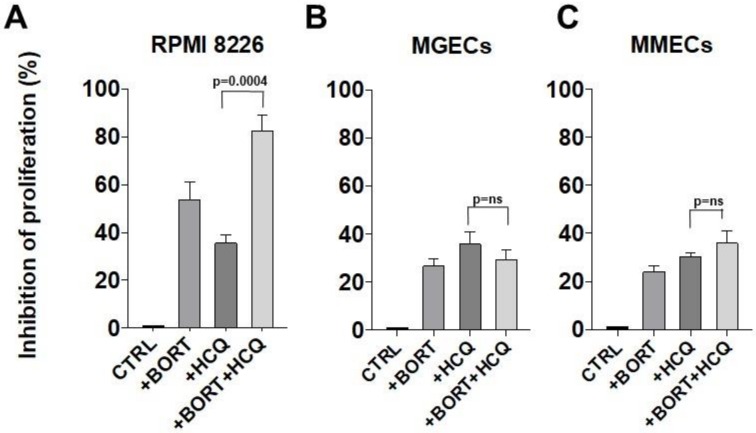
Bortezomib and HCQ differentially modulate MM cell proliferation. RPMI 8226 cells (**A**), MGECs (*n* = 6) (**B**), and MMECs (*n* = 6) (**C**) were treated with or without bortezomib (10 nM), HCQ (100 uM) or with both drugs for 24 h, after which the inhibition of proliferation was evaluated by measuring cell viability. Mann–Whitney U test. ns = not significant.

Next, we asked whether the inhibition of proteasome by bortezomib and the blockade of autophagy by HCQ affected the proliferation (and viability) of PCs and ECs in MGUS and MM patients (Figure 6). Myeloma PCs, MGECs, and MMECs were cultured in the presence of 10 nM bortezomib and 100 μM HCQ, either alone or in combination. After 24 h, proliferation was measured in a luciferin–luciferase reaction. In myeloma PCs, although bortezomib and HCQ alone inhibited proliferation compared to control cells (53.7 ± 7.06% and 35.54 ± 3.63% respectively), combination treatment caused a much stronger inhibition compared with control cells and with treatment with HCQ alone (up to 82.53 ± 6.34%; *p* = 0.0004; Figure 6A). This effect was partially lost in MGECs and MMECs (Figure 6B,C). After treatment with bortezomib and HCQ alone, proliferation was inhibited in MGECs (26.48 ± 2.93% and 35.76 ± 5.06%, respectively) (Figure 6B) and in MMECs (23.93 ± 2.6% and 30.27 ± 1.41%, respectively) (Figure 6C) with no significant differences between the treatment with HCQ alone and the treatment with both bortezomib and HCQ (Figure 6B,C).

**Figure 7:**
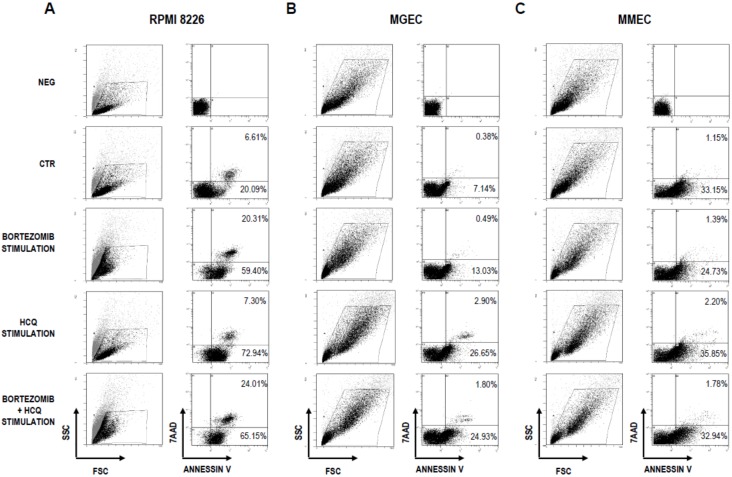
Effect of combination treatment with bortezomib and HCQ on cell cytotoxicity. Representative flow cytometry plots show changes in cytotoxicity for (**A**) RPMI 8226 cells, (**B**) MGECs (*n* = 9), and (**C**) MMECs (*n* = 11) treated with or without bortezomib (10 nM), HCQ (100 uM), or with both drugs for 24 h.

Finally, we determined the cytotoxicity of the combined treatment (bortezomib and HCQ in myeloma PCs, MGECs, and MMECs cultured in the presence of 10 nM bortezomib or 100 μM HCQ alone or in the presence of both drugs). After 24 h, cell apoptosis was evaluated by flow cytometry (Figure 7). Combined treatment resulted in the enhancement of bortezomib-induced cytotoxicity in myeloma PCs and MGECs (Figure 7A,B), whereas in MMECs the percentages of early and late apoptotic cells were largely unchanged (Figure 7C).

Overall, these findings demonstrated that myeloma PCs are more sensitive than ECs to the simultaneous blockade of autophagy and proteasome. Moreover, MGECs were more responsive than MMECs to the combination of bortezomib and HCQ, suggesting that ECs become resistant during the progression from MGUS to MM.

## 3. Discussion

Although the introduction of bortezomib as a therapeutic strategy has improved the overall survival of MM patients, from less than 3 years in the 1990s to the current rate of roughly 10 years for patients under 65 and 5–6 years for older patients [42], 15–20% of high-risk patients do not respond to bortezomib over time or become resistant to treatment and die within 2 years after the diagnosis [42,43]. The efficacy of proteasome inhibitor therapy is linked to the capacity of tumor PCs to degrade unfolded/misfolded proteins in the proteasome. Myeloma PCs are characterized by reduced proteasome expression but an increased proteasome workload (likely due to immunoglobulin (Ig) synthesis) and greater apoptotic sensitivity to bortezomib [44,45]. In the presence of a proteasome inhibitor, a potential PC survival strategy is the activation of autophagy, an alternative cellular mechanism of protein degradation [19]. Proteasome inhibition by bortezomib in tumor PCs results in the accumulation of unfolded/misfolded proteins in the endoplasmic reticulum (ER), a response referred to as ER stress, accompanied by the overproduction of reactive oxygen species in the ER, and the activation of unfolded protein cascade components, especially the protein kinase R-like (PKR-like) ER kinase, resulting in the induction of autophagy [46]. The ER-stress-induced activation of c-Jun N-terminal kinase is another possible mechanism by which bortezomib stimulates autophagy [47] in myeloma PCs as a prosurvival process. Therefore, simultaneously targeting proteasome and autophagy in MM should result in enhanced anti-myeloma effects. Vogl et al. demonstrated an enhanced antitumor efficacy by the combined targeting of proteasomal and autophagic protein degradation using bortezomib and HCQ in MM [38].

The present study provided evidence that combined treatment with bortezomib and HCQ targets not only myeloma PCs but also bone marrow ECs responsible for tumor angiogenesis, a recognized hallmark of the MGUS-to-MM progression, associated with a poor prognosis [48,49]. Our results showed that bortezomib has opposite effects on autophagy in PCs and ECs. In myeloma PCs, bortezomib interacts synergistically with HCQ resulting in high ER stress levels due to the intracellular accumulation of large amounts of toxic misfolded immunoglobulins, and predisposition to apoptosis given that the threshold for apoptosis activation was pushed to lower stress levels when autophagy was downregulated [44,50]. Interestingly, shrank autophagy correlated with permeation of permissive immune microenvironment and infiltration of regulatory T cells [51]. Given that bone milieu and endothelium plays a major role in MM progression and drug-resistant phenotype, actively modulating immune-microenvironment at the same time [52], it is tempting to speculate a pivotal role of autophagy in a vicious cycle existing between tumoral myeloma PCs and the primed permissive stromal cells [53]. In the frame of this thinking, an altered basal autophagy process could occur in myeloma PCs because of the many genetic alterations, also in a tumor microenvironment driven fashion. [54].

To corroborate this hypothesis, we used the STRING database [55,56] to investigate ‘possible’ interactions among the major proteins involved in autophagy: LC3B-I (MAP1LC3A), LC3B-II (MAP1LC3B), and SQSTM-1 (p62), the autophagy-related (ATG) family proteins involved in autophagosome formation, and the driver mutated genes in RPMI-8226 and JJN3 myeloma cell lines, Kirsten rat sarcoma viral oncogene homolog (KRAS) and MAF1, respectively. A diagram depicting the protein–protein interactions is presented in Figure S2. The gene ontology report analysis confirmed that the ATG family proteins microtubule-associated proteins 1A/1B light chain 3A (MAP1LC3A), microtubule-associated proteins 1A/1B light chain 3B or LC3 (MAP1LC3B), SQSTM-1, and Cyclin-dependent kinase inhibitor 2A (CDKN2A) are tangled in distinct autophagic processes (mitophagy, Gene Ontology (GO):0000422 and macro-autophagy, GO:0016236; blue and red sectors, respectively), in apoptosis (GO:0009267; green sectors) because of a strong connection with tumoral protein 53 (TP53) and B-cell lymphoma-2 (BCL2), and in the positive regulation of cellular metabolic processes because of interactions with TP53, BCL2, MYC, epidermal growth factor receptor 2 (EGFR2), erythroblastic oncogene B2 (ERBB2), KRAS, and MAF1 (GO:0031325, yellow sectors). Collectively, the generated protein–protein interaction analysis indicated a significant correlation between the driver oncogenomic mutation KRAS and MAF1 and abnormal basal autophagy in myeloma PCs. Therefore, these results suggest a plausible hypothesis that prompts further validation on a statistically powered study. Remarkably, abnormal basal autophagy was absent in ECs in which the effect of bortezomib contrasted with that of HCQ and resulted in an increase of the autophagic flux rather than in autophagy blockade, with evidence of increased autophagosome formation at microscopy, events associated with EC survival, and increase of proliferation. Ongoing and planned studies will further address the possibility of better elucidating and fingerprinting autophagy marker levels in a dynamic fashion to accurately assess changes during the different steps of the autophagy process in any given biological setting.

Overall, our data show that while combination treatment with bortezomib and HCQ significantly downregulates autophagy in myeloma PCs promoting PC death, it also positively modulates autophagy in ECs, supporting their survival. Thus, autophagy inhibitors, such as HCQ, when used in association with bortezomib, may induce this response in resistant myeloma PCs, although an anti-angiogenic drug must be included to achieve a complete remission, as the simultaneous pro-angiogenic effect could, in fact, promote the proliferation of a small residual tumor PC clone to cause relapse.

Accordingly, our study should be followed by further translational investigations of the role of therapeutic suppression in autophagy modulation. Statistically powered clinical studies aimed at identifying the therapeutic relevance of this biologic network will contribute to the elucidation of the drug resistance mechanisms arising from these molecular events.

## 4. Materials and Methods

### 4.1. Study Subjects and Biological Samples

Bone marrow samples were obtained from 20 patients with newly diagnosed monoclonal gammopathies. Patients were classified as having MGUS (*n* = 9) or symptomatic MM (*n* = 11) according to the International Myeloma Working Group criteria [57]. Bone marrow sampling consisted of aspiration followed by biopsy of the posterior iliac crest. The study protocol was approved by the University of Bari Medical School Ethics Committee and conformed to the good clinical practice guidelines of the Italian Ministry of Health. Written informed consent was obtained from each patient in accordance with the Declaration of Helsinki.

### 4.2. Plasma Cell Lines

RPMI-8226 and JJN-3 human MM cell lines and HUVECs were purchased from the ATCC. The MM cell lines were cultured in RPMI-1640 supplemented with 10% fetal bovine serum (FBS), 2 mM L-glutamine, 100 U penicillin/mL, and 100 µg streptomycin/mL (all from Sigma-Aldrich, St. Louis, MO, USA). HUVECs were cultured in endothelial cell growth medium supplemented with 5% FBS, cytokines, and growth factors (EGM-2, Lonza, Basel, Switzerland). All cells were grown at 37 °C in a humidified atmosphere containing 5% CO_2._

### 4.3. Primary Endothelial Cell Preparation

Bone marrow mononuclear cells (BMMCs) were isolated by Ficoll-Paque Plus (GE Healthcare Life Sciences, Björkgatan, Sweden) density gradient centrifugation. ECs were isolated by automated magnetic cell sorting using anti-CD31 microbeads (Miltenyi Biotec, Bergisch Gladbach, Germany) from adherent BMMCs that had been cultured for 3 weeks in Dulbecco’s modified Eagle’s medium supplemented with 10% FBS, 2 mM L-glutamine, 100 U penicillin/mL, and 100 μg streptomycin/mL (all from Sigma-Aldrich). All sorted cell populations exhibited >95% purity, as determined by flow cytometry of immunostained cells. Purified ECs were grown and expanded for four passages in fibronectin-coated culture dishes (Becton Dickinson-BD, San Jose, CA, USA) in EGM-2.

### 4.4. Reagents

Bortezomib (Velcade, Selleckchem, USA) was purchased from Selleckchem and used at a concentration of 10 nM. HCQ was purchased from Sigma-Aldrich and used at a concentration of 100 μM.

### 4.5. Immunoblotting

ECs (5 × 10^5^) or PCs (1 × 10^6^) cultured with bortezomib and/or HCQ as previously described were harvested, washed, and lysed using radioimmunoprecipitation assay (RIPA) lysis buffer (Sigma-Aldrich, St. Louis, Missouri, USA) supplemented with protease inhibitors (Sigma-Aldrich, St. Louis, Missouri, USA). The cell lysates (35 μg) were subjected to SDS-PAGE and transferred to a nitrocellulose membrane (Bio-Rad, Hercules, USA). After blocking with 5% dry milk, the membrane was incubated overnight at 4 °C with anti-microtubule-associated protein 1 light chain 3 beta (LC3B; Cell Signaling, Leiden, The Netherlands), anti-sequestosome-1/ubiquitin-binding protein p62 (SQSTM1/p62; Abcam, Cambridge, United Kingdom), or anti-β-actin (Sigma-Aldrich, St. Louis, Missouri, USA) primary antibodies, followed by a washing step and a 1-h incubation with the appropriate Horseradish Peroxidase (HRP)-conjugated secondary antibody. ECL detection was performed using the Clarity™Western ECL blotting substrate (BioRad, Hercules, USA) and subsequent quantification with the ChemiDoc™ XRS+ system (Image Lab™ Software) (Biorad, Hercules, USA).

### 4.6. Cytotoxicity Assay

PCs (2 × 10^4^) or immunomagnetically purified ECs (1.5 × 10^4^) were cultured in 96-well round plates in the presence of bortezomib (0.5–1000 nM) and/or HCQ (0.5–500 µM) for 2, 4, 6, and 24 h. The cells were grown in 200 μL of RPMI-1640 or EGM-2 medium at 37 °C in a humidified atmosphere containing 5% CO_2_. Cytotoxicity was determined using the CellTiter-Glo^®^ luminescent cell viability assay kit (Promega, Woods Hollow Road Madison, USA). The luminescence signal was detected using a VICTOR multilabel plate reader.

### 4.7. Apoptosis Assay

PCs (3 × 10^5^) or ECs (5 × 10^5^) were cultured in 60-mm dishes alone or in the presence of 10 nM bortezomib and/or 100 μM HCQ. After 24 h, the cells were harvested, washed once with 1 × Annexin V binding buffer, incubated with 7-ADD and PE-Annexin V (all from Becton Dickinson-BD, San Jose, CA, USA), and immediately analyzed by flow cytometry using an FC500 flow cytometer and CXP software (Beckman Coulter, USA).

### 4.8. Protein–Protein Interaction Analysis

Gene ontology analysis was performed by uploading peptides involved in the autophagy pathway to the Search Tool for the Retrieval of Interacting Genes (STRING) (http://string-db.org) (D. Szklarczyk D561).

### 4.9. Statistical Analysis

Statistical analyses were performed using Prism (GraphPad Software, San Diego, CA, USA). Parametric statistics were applicable because of the normally distributed data. Tests included an unpaired *t*-test for the comparisons of groups. *p*-values are shown only for statistically significant comparisons. A *p* value < 0.05 was considered to indicate statistical significance.

## Figures and Tables

**Table 1:** Abbreviations and acronyms.

Abbreviation	Full Form and Definition
MM	Multiple myeloma
MGUS	Monoclonal gammopathy of undetermined significance
PCs	Plasma cells
HCQ	Hydroxychloroquine
ECs	Endothelial cells
MGECs	Endothelial cells from MGUS patients
MMECs	Endothelial cells from MM patients
BMMCs	Bone marrow mononuclear cells
ATG	Autophagy related
MAP1LC3A	Microtubule-associated proteins 1A/1B light chain 3A
MAP1LC3B	Microtubule-associated proteins 1A/1B light chain 3B or LC3
LC3B	Microtubule-associated protein light chain beta
LC3-I	Cytosolic form of LC3
LC3-II	Membrane-bound form of LC3, phosphatidylethanolamine conjugated
RPMI 8226	Human myeloma cell line
JJN3	Human myeloma cell line
HUVECs	Human umbilical vein endothelial cells
ER	Endoplasmic reticulum
PKR-like	Protein kinase R-like
STRING	Biological database and web resource of known and predicted protein–protein interactions
SQSTM-1	Sequestosome-1 also known as p62
KRAS	Kirsten rat sarcoma viral oncogene homolog
MAF1	Repressor of RNA polymerase III transcription
CDKN2A	Cyclin-dependent kinase Inhibitor 2A
TP53	Tumoral protein 53
BCL2	B-cell lymphoma 2. Apoptosis regulator
MYC	Family of regulator genes and proto-oncogenes that code for transcription factors
EGFR2	Epidermal growth factor receptor 2
ERBB2	Erythroblastic oncogene B2. Receptor tyrosine kinase
RAF-1	Proto-oncogene, serine/threonine kinase

## Supplementary Materials

The following are available online at https://www.mdpi.com/2077-0383/9/2/552/s1, Figure S1: Autophagy saturation with HCQ in PCs, MGECs and MMECs; Figure S2: Protein-protein interaction network obtained from STRING database.
