# Supplementary material for: Bortezomib Treatment Modulates Autophagy in Multiple Myeloma
Source: J Clin Med. 2020 Feb 18;9(2):552. doi: 10.3390/jcm9020552 (PMC7073518; doi:10.3390/jcm9020552)
Supplement: Supplementary file 1 [file jcm-09-00552-s001.pdf]

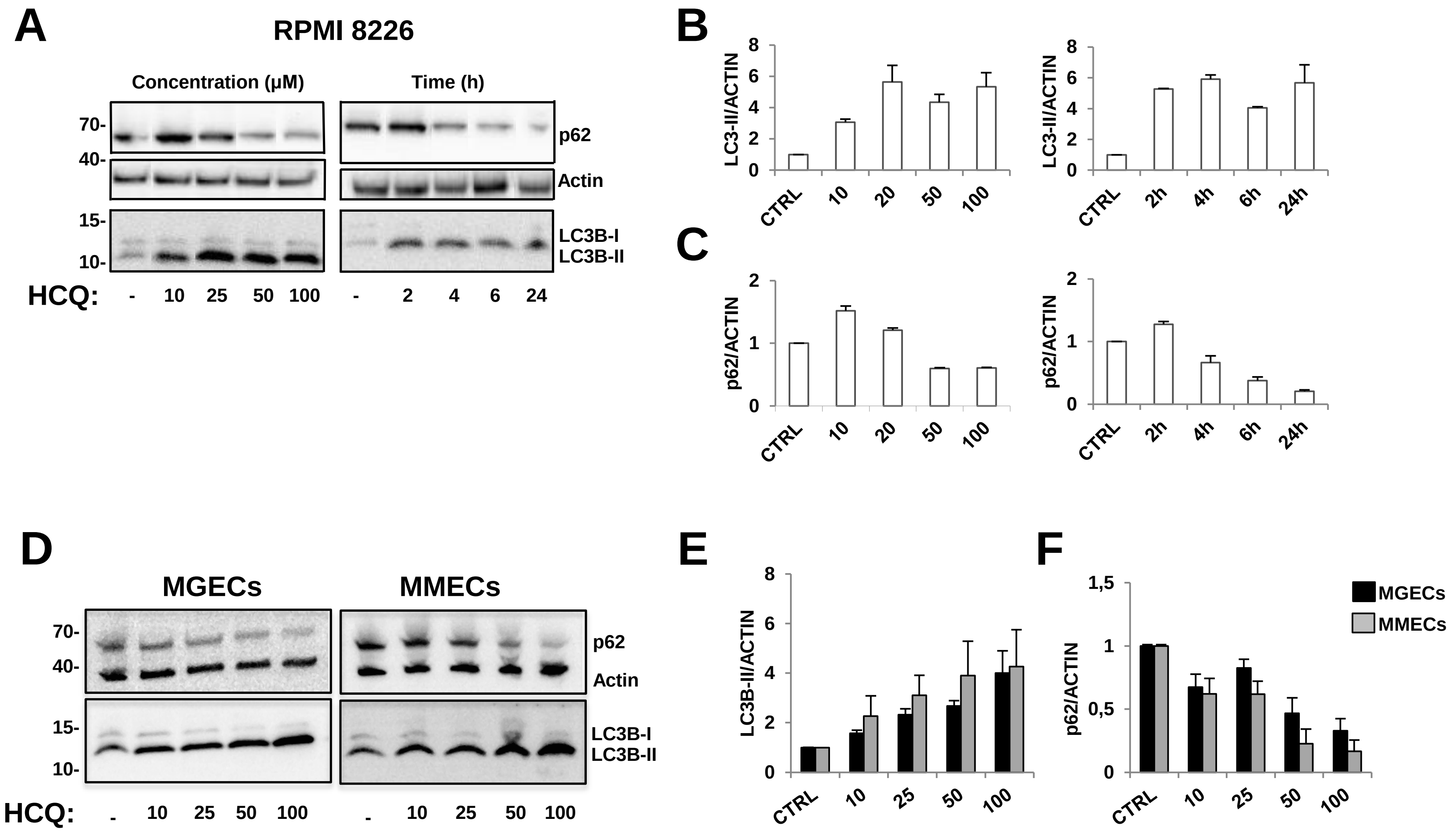


Supplemental Fig. 1

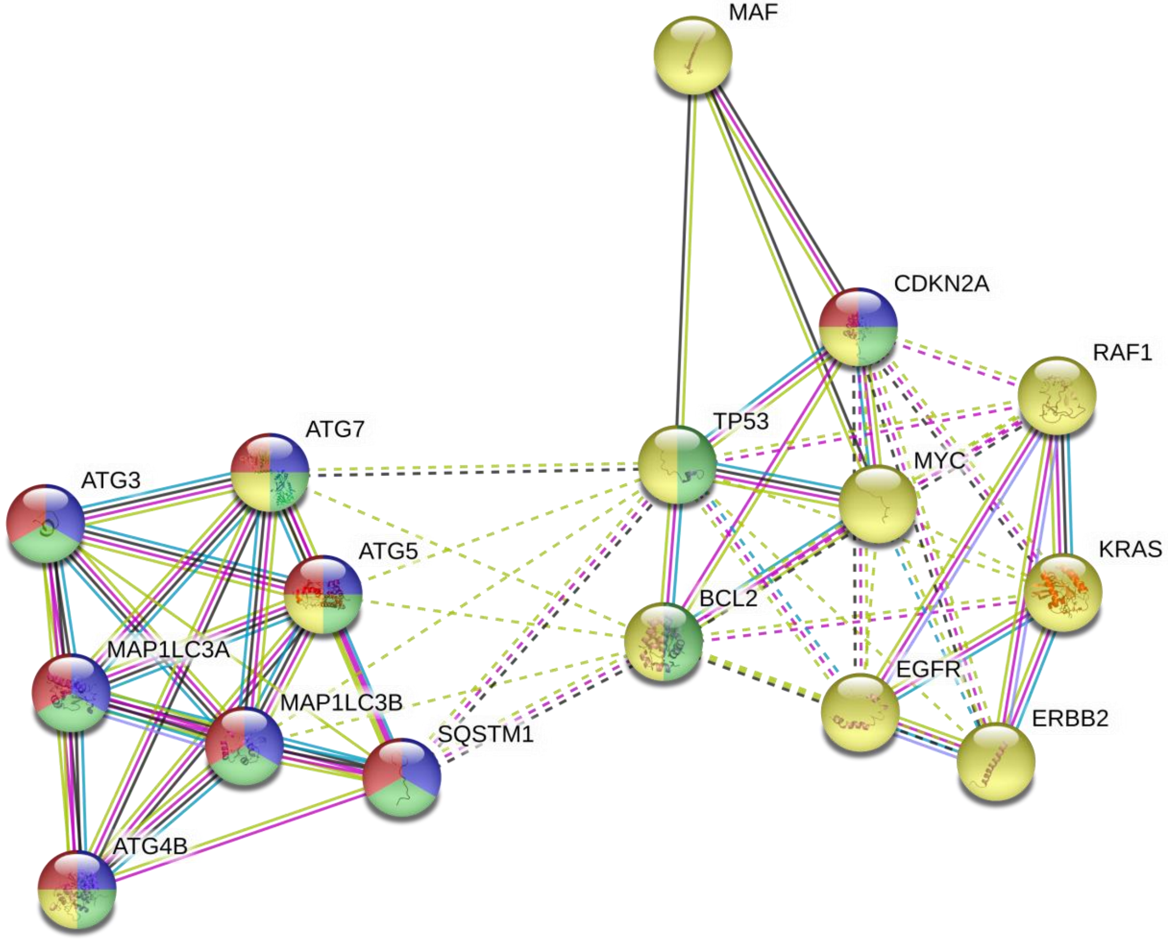


**Supplemental Fig. 2**

**Supplemental Figure 1**. Autophagy saturation with HCQ in PCs, MGECs and MMECs. (**A, D**) Western blot analysis to determine the optimal concentration of HCQ and duration of incubation to achieve autophagy saturation in RPMI 8226 cells (**A**) and in MGECs and MMECs (**D**). The cells were incubated with HCQ at the indicated concentrations and times, followed by immunoblotting for the expression of LC3B-II, actin, and p62. (**B-F**) Densitometric analysis of RPMI 8226 (**B-C**), MGEC (black bars), and MMEC (gray bars) (**E-F**) lysates for LC3B-II (**B, E**) and p62 (**C, F**) expression. The results are expressed as the fold-change normalized to the β-actin level and relative to the control

**Supplemental Figure 2.** Protein-protein interaction network obtained from STRING database. Sixteen proteins were interrogated from the database to be related with autophagy and with the mutated gene encoding proteins KRAS and MAF1. Colors of edges refer to the type of evidence linking the corresponding proteins: mitophagy, blue sectors, 1% False discovery rate (FDR) 1.76e-14; macroautophagy, red sectors; FDR 1.39e-9; apoptosis (cellular response to starvation), green sectors FDR 1.216e-07 and positive regulation of cellular metabolic processes, yellow sectors. String analysis of differentially expressed proteins.

Abbreviations: MAP1LC3A = microtubule Associated Protein 1 Light Chain 3 Alpha; SQSTM-1 = sequestosome-1; KRAS = kirsten rat sarcoma viral oncogene homolog; CDKN2A = cyclin-dependent kinase Inhibitor 2A; TP53 = tumoral protein 53; ATG = autophagy-related proteins; TP53 = tumoral protein 53; BCL2 = B-cell lymphoma 2; EGFR2 = epidermal growth factor receptor 2; ERBB2 = erythroblastic oncogene B2.
